# The core and unique proteins of haloarchaea

**DOI:** 10.1186/1471-2164-13-39

**Published:** 2012-01-24

**Authors:** Melinda D Capes, Priya DasSarma, Shiladitya DasSarma

**Affiliations:** 1 Department of Microbiology and Immunology, Institute of Marine and Environmental Technology, University of Maryland, 701 East Pratt Street, Baltimore, MD 21202 USA

## Abstract

**Background:**

Since the first genome of a halophilic archaeon was sequenced in 2000, biologists have been advancing the understanding of genomic characteristics that allow for survival in the harsh natural environments of these organisms. An increase in protein acidity and GC-bias in the genome have been implicated as factors in tolerance to extreme salinity, desiccation, and high solar radiation. However, few previous attempts have been made to identify novel genes that would permit survival in such extreme conditions.

**Results:**

With the recent release of several new complete haloarchaeal genome sequences, we have conducted a comprehensive comparative genomic analysis focusing on the identification of unique haloarchaeal conserved proteins that likely play key roles in environmental adaptation. Using bioinformatic methods, we have clustered 31,312 predicted proteins from nine haloarchaeal genomes into 4,455 haloarchaeal orthologous groups (HOGs). We assigned likely functions by association with established COG and KOG databases in NCBI. After identifying homologs in four additional haloarchaeal genomes, we determined that there were 784 core haloarchaeal protein clusters (cHOGs), of which 83 clusters were found primarily in haloarchaea. Further analysis found that 55 clusters were truly unique (tucHOGs) to haloarchaea and qualify as signature proteins while 28 were nearly unique (nucHOGs), the vast majority of which were coded for on the haloarchaeal chromosomes. Of the signature proteins, only one example with any predicted function, Ral, involved in desiccation/radiation tolerance in *Halobacterium *sp. NRC-1, was identified. Among the core clusters, 33% was predicted to function in metabolism, 25% in information transfer and storage, 10% in cell processes and signaling, and 22% belong to poorly characterized or general function groups.

**Conclusion:**

Our studies have established conserved groups of nearly 800 protein clusters present in all haloarchaea, with a subset of 55 which are predicted to be accessory proteins that may be critical or essential for success in an extreme environment. These studies support core and signature genes and proteins as valuable concepts for understanding phylogenetic and phenotypic characteristics of coherent groups of organisms.

## Background

Extremely halophilic Archaea (haloarchaea) have adapted to thrive in environments of high salinity, desiccation, and intense solar radiation. These microorganisms require at least 1.5 - 2.5 M NaCl for viability and typically display optimal growth in NaCl concentrations at or above 3.5 M. Haloarchaea commonly inhabit hypersaline environments, e.g. salt lakes, salterns, and heavily salted hides, meats, fish, and sauces [1-3]. Additionally, haloarchaea have been shown to survive space conditions [4] and viable cells have been reported from ancient deep underground salt deposits [5,6]. Unlike most other extremophilic and archaeal microorganisms, haloarchaea form a monophyletic and coherent taxonomic group, the family Haloarchaeaceae [7].

The *Halobacterium *sp. NRC-1 genome sequence gave researchers the first opportunity, at the genome level, to probe the mechanisms of adaptation to hypersaline brine [8,9]. Characterization of the 2 Mbp chromosome and two large megaplasmids showed that the overwhelming majority of predicted proteins were highly acidic, with a pI mode of 4.2, and very few neutral or basic proteins [10,11]. In contrast, predicted proteins from most other non-haloarchaeal and bacterial organisms had equal fractions of acidic and basic components. The negatively charged residues in haloarchaeal proteins were predominantly found at the protein surface and predicted to function in enhancing their solubility and stability in high salt concentrations. A few individual haloarchaeal proteins have been crystallized, e.g. malate dehydrogenase, dihydrofolate reductase, and DNA sliding clamp (PCNA), and they all display markedly more acidic residues than non-haloarchaeal homologs. They also possess clusters of negative charges on the surface [12-14]. The high prevalence of negatively charged surface residues produces tightly bound hydration shells with salt ions bound at the protein surface [16,17].

Several previous studies have examined the gene content in haloarchaea, including one aimed at identifying information transfer genes and another concerning metabolic genes [18,19]. While a significant degree of conservation was found among the essential components of DNA replication, repair, and recombination, transcription, and translation, the study of metabolic genes showed substantially more diversity. Indeed, this diversity was illustrated by the recent identification of genes for a new pathway in central carbon metabolism, the methylaspartate cycle, in several haloarchaea [20]. An additional characteristic observed in most haloarchaeal genomes is the presence of large megaplasmids or minichromosomes which often harbor important or essential genes [21]. Gene content in these large extrachromosomal elements was compared and resulted in the finding of expanded gene families for replication and transcription initiation, e.g. *orc *and *tfb *[18], as well as the presence of a variety of genes needed for cell survival, e.g. an amino-acyl tRNA synthetase [9], resistance to arsenic [22], and production of buoyant gas vesicles [9].

**Figure 1:**
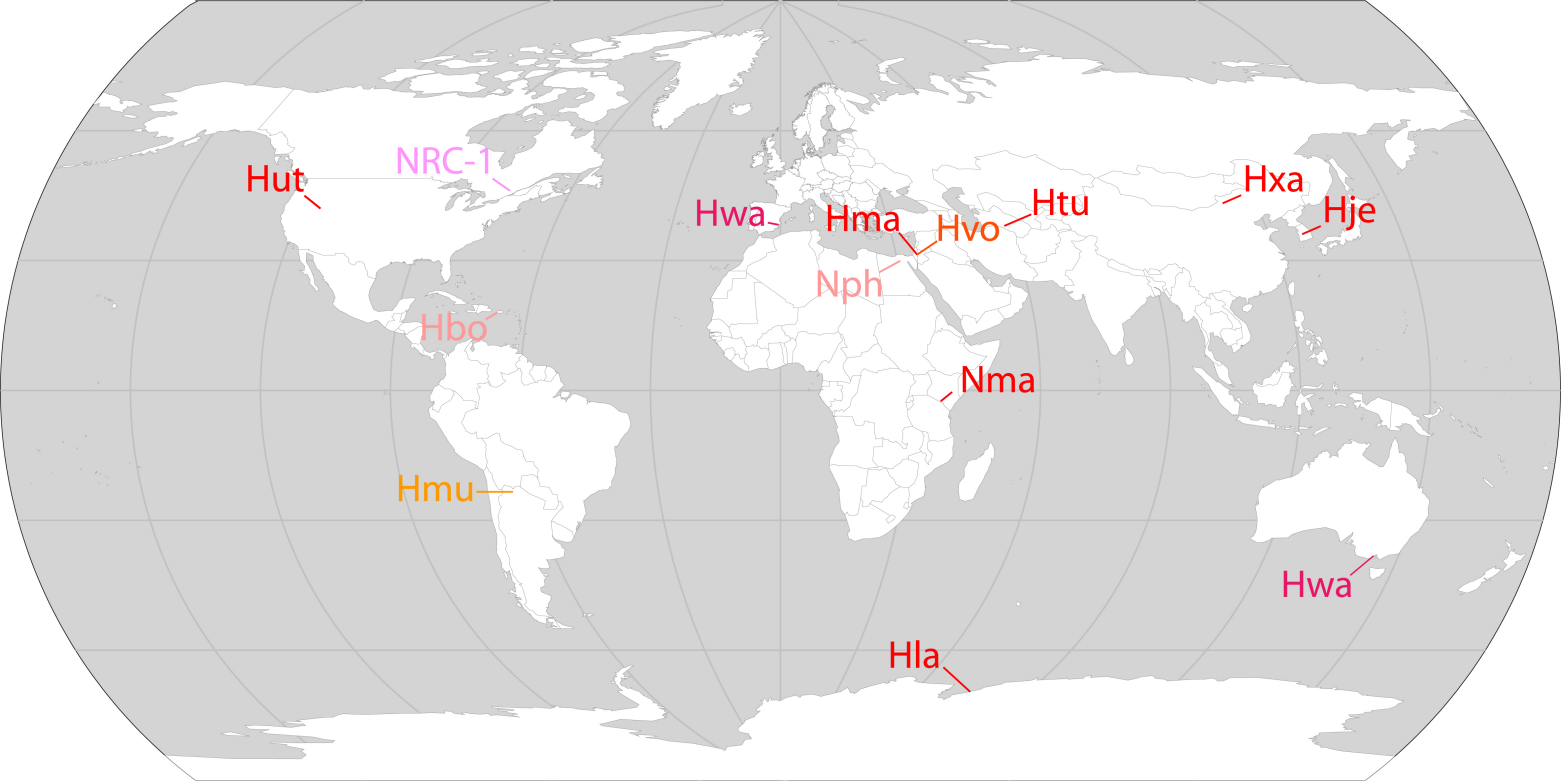
**World map showing the locations of isolation for haloarchaeal organisms with sequenced genomes**. The organisms represent a significant geographical diversity of haloarchaeal isolates: [*Halobacterium *sp. NRC-1 (NRC-1), the model haloarchaeal organism isolated from salted food in Canada, *Haloarcula marismortui *(Hma), a physiologically versatile extreme halophile from the Dead Sea, *Natronomonas pharaonis *(Nph), an alkaliphilic extreme halophile from an Egyptian soda lake, *Haloquadratum walsbyi *(Hwa), a square-shaped extreme halophile from solar salterns in Australia and Spain, *Halorubrum lacusprofundi *(Hla), a cold-adapted halophile from an Antarctic lake, *Halogeometricum borinquense *(Hbo), a pleomorphic extreme halophile from a solar saltern in Puerto Rico, *Halomicrobium mukohataei *(Hmu), a rod-shaped halophile from an Argentinean salt flat, *Halorhabdus utahensis *(Hut), a pleomorphic extreme halophile from sediments of the Great Salt Lake, USA, *Haloferax volcanii *(Hvo), a moderate halophile from Dead Sea mud, *Haloterrigena turkmenica *(Htu), a pleomorphic halophile from Turkmenistan, *Natrialba magadii *(Nma), an alkaliphilic halophile from Lake Magadi, Kenya, *Halalkalicoccus jeotgali *(Hje), extreme halophile from Korean fermented seafood, and *Halopiger xanaduensis *(Hxa), extreme halophile from saline Lake Shangmatala, China. Labels are based on the color of haloarchaeal colonies.

In the current study, we present a comprehensive analysis of haloarchaeal genomes aimed at identifying the core haloarchaeal proteins and uniquely haloarchaeal groups. Halophilic Archaea representing thirteen different genera were included, all within the Haloarchaeaceae family. These microorganisms represent both geographic and phylogenetic diversity, including isolates from all 7 continents (Figure 1) and almost half of the genera in this tight clade of the Euryarchaea [2]. The genome-wide analysis produced nearly 800 protein clusters that are completely conserved among sequenced haloarchaea and a subset of 55 protein families that are unique to this family of extremophilic microbes.

## Results

### Haloarchaeal orthologous groups (HOGs)

**Figure 2:**
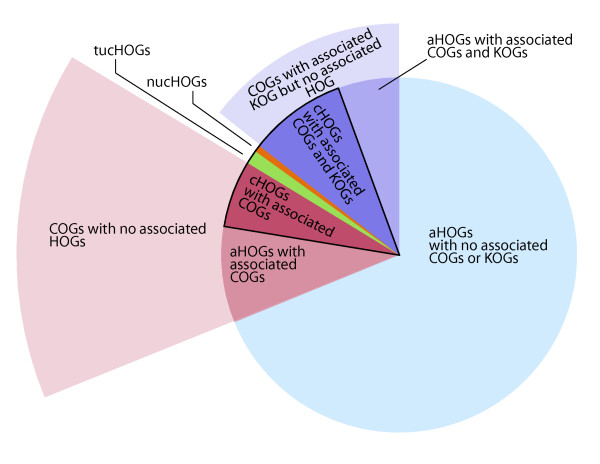
**Venn diagram showing the distribution and relationship among clusters of orthologous groups for haloarchaea (HOGs), prokaryotes (COGs), and eukaryotes (KOGs)**. Accessory HOGs (aHOGs) and core HOGs (cHOGs) (black outline) were associated with COGs and KOGs (drawn to scale). cHOGs not associated with COGs or KOGs were termed truly unique cHOGs (tucHOGs) or nearly unique cHOGs (nucHOGs). COGs and associated KOGs with no associated HOG are illustrated for comparison.

Using the best reciprocal hit method, 31,312 predicted proteins from nine complete haloarchaeal genomes (*Halobacterium *sp. NRC-1, *Haloarcula marismortui*, *Natronomonas pharaonis*, *Haloquadratum walsbyi*, *Halorubrum lacusprofundi*, *Halogeometricum borinquense*, *Halomicrobium mukohataei*, *Halorhabdus utahensis*, and *Haloferax volcanii*) were initially compared to form 4,455 *haloarchaeal orthologous groups *(HOGs) (see Table 1 and Table 2; Figure 1 and 2) [23,24]. Our results showed that the overwhelming majority of predicted haloarchaeal proteins were members of HOGs, ranging from a high of 82.8% for *Halobacterium *sp. NRC-1 to a low of 73.9% for *H. utahensis*. These results underscored the close relationship of these haloarchaeal species.

**Table 1:** Definition of proteins clusters.

Protein clusters	Description	Reference
COG	Clusters of orthologous groups in 26 or 66 microorganisms*	[23,24]
KOG	Clusters of orthologous groups in 7 eukaryotic organisms*	[24]
arCOG	Clusters of orthologous groups in 41 or 70 archaeal microorganisms	[28]
HOG	Clusters of orthologous groups in 13 haloarchaeal microorganisms	This work
cHOG	Conserved orthologous groups in all 13 haloarchaeal microorganisms	This work
aHOG	HOGs not conserved in all 13 haloarchaeal microorganisms	This work
ucHOG	cHOGs not associated with any COGs or KOGs	This work
tucHOG	ucHOGs that do not have any homologs among any other proteins	This work
nucHOG	ucHOGs that have 1 or more non-haloarchaeal homologs	This work

*COGs and KOGs both include *S. cerevisiae*

**Table 2:** Nine haloarchaeal organisms used to identify HOGs.

Genome	Proteome size	Clustered proteins	Core proteome
*Halobacterium *sp. NRC-1	2626	2174 (82.8%)	857 (32.6%)
*Haloarcula marismortui *	4240	3464 (81.7%)	893 (23.1%)
*Natronomonas pharaonis *	2822	2285 (81.0%)	847 (30.0%)
*Haloquadratum walsbyi *	2626	2108 (80.3%)	835 (31.8%)
*Halorubrum lacusprofundi *	3913	3166 (80.9%)	870 (22.2%)
*Halogeometricum borinquense *	4303	3209 (74.6%)	891 (20.7%)
*Halomicrobium mukohataei *	3548	2902 (81.8%)	858 (24.2%)
*Halorhabdus utahensis *	3160	2334 (73.9%)	856 (27.1%)
*Haloferax volcanii *	4074	3240 (79.5%)	870 (21.4%)

### Core HOGs (cHOGs)

**Figure 3:**
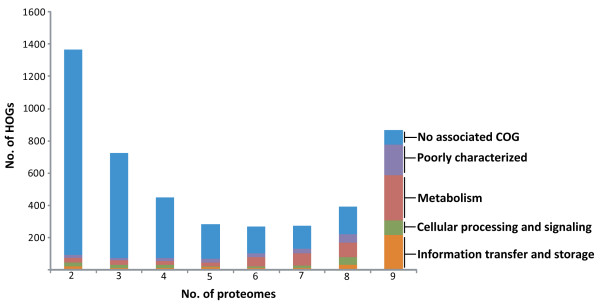
**Functional classification of haloarchaeal orthologous groups (HOGs) for nine haloarchaea**. Predicted functions were assigned to core (9 genomes) and accessory (2 - 8 genomes) HOGs based on association with COGs. Several HOGs were associated with one or more COG and all predicted functions are illustrated. Based on predicted functions, HOGs were classified as likely involved in information transfer and storage (orange), cellular processing and signaling (green), or metabolism (red). Predicted functions could not be assigned to HOGs associated with poorly characterized COGs (purple) or with no associated COG (blue).

We examined the abundance of the haloarchaeal proteins present in these 4,455 HOGs and found a bimodal distribution (Figure 3). The largest number of protein clusters were found in either 2 or 3 haloarchaea (1358 or 716, respectively) or all 9 members (799 protein clusters), and the protein clusters with an intermediate (4 - 8) number of haloarchaea were less abundant (250 - 442). The 799 clusters conserved in all nine organisms were designated as *core *haloarchaeal orthologous groups (cHOGs) (see Additional file 1) and represented proteins that are known or expected to be important or essential in all of the haloarchaea (see below). Taking into account that several HOGs correspond to more than a single COG and KOG, comparison of the cHOGs to the COG and KOG databases in NCBI showed that of the 799 cHOGs, 422 corresponded to both COGs and KOGs and 288 corresponded to COGs only, with 89 novel clusters unique to haloarchaea.

### Uniquely haloarchaeal orthologous groups (ucHOGs, tucHOGs, and nucHOGs)

Of the 799 cHOGs present in all nine haloarchaea, 89 (11%) appeared to be unique to haloarchaea based on their absence in both the COG and KOG databases. These *unique *core HOGs (ucHOGs) were candidates for being 'signature' proteins for this clade, based on their ubiquity among haloarchaea and absence in non-haloarchaeal clades (Figure 2). However, since the members of these protein clusters were quite diverse, with the percent identity varying widely (between 22% and 85%), we re-appraised the statistical significance of group members by carrying out pairwise alignments of the proteins within each cluster, including randomized global alignments for statistical analysis using the Needleman and Wunsch algorithm [25,26]. Using this approach, we were able to establish a 99.9999% confidence level for pairs of sequences among proteins within each cluster.

With the rapid sequencing of new haloarchaeal genomes, we further scrutinized the 89 ucHOGs using a sequential multi-step approach: (1) protein sequences were BLASTed against four recently available complete haloarchaeal genome sequences (*Haloterrigena turkmenica*, *Natrialba magadii*, *Halalkalicoccus jeotgali*, and *Halopiger xanaduensis*) to find conserved haloarchaeal homologs, (2) protein sequences were BLASTed against the NCBI non-redundant database to find non-haloarchaeal hits, and (3) any non-haloarchaeal hits identified were aligned with each member of the cHOG cluster. Of the 89 clusters with no associated COGs or KOGs, all members of 55 ucHOG clusters were found to be *truly *unique core haloarchaeal orthologous groups and named tucHOGs (Figure 2). Of the remaining 34 clusters, 6 were absent in one or more of the four newly sequenced genomes, and 29 had one or more members with at least one hit to a non-haloarchaeal peptide. Proteins from six clusters had hits to over a dozen different non-haloarchaeal proteins and proteins from the remaining 23 clusters had fewer hits, ranging from 1 - 10 per cluster. The significance of hits was evaluated by base composition-preserved randomized alignments. This analysis showed that the 28 cHOG clusters with hits to non-haloarchaeal proteins were not entirely unique to the haloarchaea with a 99.0% or higher level of confidence, and were named *nearly *unique core haloarchaeal orthologous groups, or nucHOGs (Figure 2).

### Genomic locations and functions of ucHOGs

**Figure 4:**
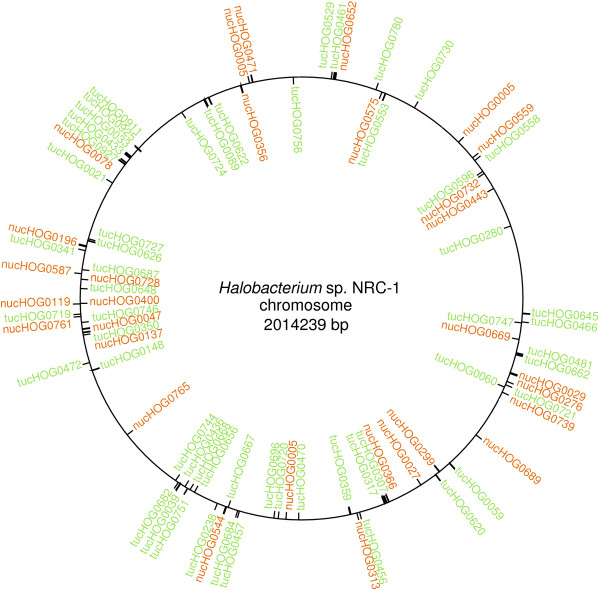
**Location of uniquely core haloarchaeal orthologous genes, or ucHOGs, coded on the chromosome of *Halobacterium *sp. NRC-1**. Map of *Halobacterium *sp. NRC-1 chromosome indicating the location of the 55 tucHOG (green), and 28 nucHOG (orange) genes. The nucHOG0027 gene present on the inverted repeat regions of pNRC100 and pNRC200 in *Halobacterium *sp. NRC-1 is not shown.

Consistent with a critical role in the biology of haloarchaea, ucHOGs were found to be encoded overwhelmingly on the main chromosomes of haloarchaeal organisms. Indeed, in five, *Halobacterium *sp. NRC-1, *H. marismortui*, *N. pharaonis*, *H. utahensis*, and *H. walsby*i, all of the tucHOG polypeptides were chromosomally encoded and dispersed relatively evenly over the entire chromosome (Figure 4). Only seven tucHOG protein genes did not map to large chromosomal replicons, with two on the small chromosome in *H. lacusprofundi*, one each on the pHB200 and pHB500 megaplasmids in *H*. *borinquense*, two on the pHV4 megaplasmid in *H. volcanii*, and one on the pHM61 megaplasmid in *H*. *mukohataei *(see Additional file 1). Similarly, all of the nucHOG proteins mapped to the large chromosomes of *N. pharaonis*, *H. walsbyi*, *H*. *borinquense, H*. *mukohataei, H. utahensis*, and *H. lacusprofundi*. A single nucHOG protein is coded on both the smaller chromosome II and pNG600 in *H. marismortui*, two nucHOGs are coded on pHV4 in *H. volcanii*, and one nucHOGs is found on the common inverted repeats of pNRC100 and 200 of *Halobacterium *sp. NRC-1 (see Additional file 1).

**Figure 5:**
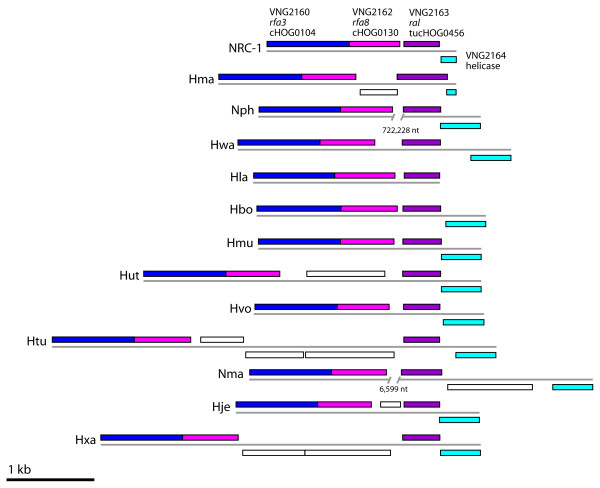
**Shared synteny of the tucHOG0456 (*ral*) gene region among the haloarchaeal chromosomes**. In *Halobacterium *sp. NRC-1, *ral *(purple) is transcriptionally linked to *rfa3 *(blue) and *rfa8 *(pink), an additional gene, coding for a predicted helicase (turquoise), near the *rfa3*-*rfa8*-*ral *operon in *Halobacterium *sp. NRC-1 is also highly conserved. Haloarchaeal chromosomes are designated with the following abbreviations: *Halobacterium *sp. NRC-1 (NRC-1), *H. marismortui *(Hma), *N. pharaonis *(Nph), *H. walsbyi *(Hwa), *H. lacusprofundi *(Hla), *H. borinquense *(Hbo), *H. mukohataei *(Hmu), *H. utahensis *(Hut), *H. volcanii *(Hvo), *H. turkmenica *(Htu), *N. magadii *(Nma), *H. jeotgali *(Hje), and *H. xanaduensis *(Hxa).

The function of only a single uniquely conserved haloarchaeal orthologous protein gene, vng2163 (cluster tucHOG0456), has so far been investigated in any detail [27]. In *Halobacterium *sp. NRC-1, the gene coding for this protein was annotated as *ral *(*rfa*-linked) due to its transcriptional linkage to two genes, *rfa3 *and *rfa8*, which encode eukaryotic replication protein A (RPA)-like single-stranded DNA binding protein subunits [27]. The genes around *ral *showed a significant degree of synteny among the haloarchaeal genomes (Figure 5), consistent with a conserved function in haloarchaea.

### Functional classification of HOGs

Biological functional categories were assigned to HOGs by membership of *Halobacterium *sp. NRC-1 HOG proteins in COGs, where possible (Table 3; Figure 3). However, the majority (86%) of *accessory *HOGs (aHOGs), protein clusters with peptide sequences from eight or fewer haloarchaea, were not members of any COGs or KOGs, or were members of poorly characterized COGs and could not be assigned to a functional class. Of the aHOGs that could be assigned functions based on COG-association, 3% were classified as being involved in information transfer and storage, or in cellular processing and signaling, and 8% were classified as being involved in metabolism.

**Table 3:** Distribution of haloarchaeal protein clusters (HOGs) among functional categories†.

	No. of Genomes							
	2	3	4	5	6	7	8	9
**no COG**	**1276**	**650**	**375**	**215**	**164**	**140**	**168**	**89**
**Information Transfer and Storage**	**20**	**13**	**12**	**14**	**10**	**11**	**31**	**217**
Translation, ribosomal structure and biogenesis	2	4	2	2	1	4	10	108
RNA processing and modification				1				
Transcription	7	5	4	6	3	5	10	49
Replication, recombination and repair	11	4	6	5	6	2	11	57
Chromatin structure and dynamics								3
**Cellular Processes and signaling**	**26**	**17**	**18**	**7**	**11**	**15**	**45**	**89**
Cell cycle control, cell division, chromosome partitioning	4	1		1		2	3	4
Defense mechanisms	6	2	2	1		2	1	5
Signal transduction mechanisms	7	2	7	2	2	2	8	8
Cell wall/membrane/envelope biogenesis	5	7	3		2	1	11	13
Cell motility	2	2	3		2	2	12	1
Intracellular trafficking, secretion, and vesicular transport		1		1		1	6	14
Posttranslational modification, protein turnover, assembly	2	2	3	2	5	5	4	44
**Metabolism**	**26**	**28**	**25**	**26**	**57**	**74**	**94**	**283**
Energy production and conversion	3	4	9	6	7	12	20	51
Carbohydrate transport and metabolism	7		2	5	3	3	12	17
Amino acid transport and metabolism	7	10	8	5	13	13	12	66
Nucleotide transport and metabolism	1	2	1	1		3	3	46
Coenzyme transport and metabolism	2				4	10	24	51
Lipid transport and metabolism	1	2		2	8	14	9	16
Inorganic ion transport and metabolism	4	7	3	5	18	16	11	22
Secondary metabolites biosynthesis, transport and catabolism	1	3	2	2	4	3	3	14
**Poorly characterized**	**18**	**17**	**17**	**20**	**26**	**32**	**52**	**190**
General function prediction only	11	9	12	16	17	22	36	114
Function unknown	7	8	5	4	9	10	16	76

†- Several HOGs were associated with one or more COG and all functional categories were tabulated.

In contrast, the great majority (89%) of cHOGs was associated with one or more COGs and KOGs, and a large fraction, 69%, was assigned to a functional class based on this criterion.

(i) Among cHOGs, we classified 25% of the protein clusters as being involved in information transfer and storage [18]. Half of the proteins in these cHOGs were involved in translation, ribosomal structure, and biogenesis, including 25 50S ribosomal subunit clusters (cHOG0202, cHOG0218, cHOG0230, cHOG0241, cHOG0248, cHOG0414, cHOG0415, cHOG0438, cHOG0478, cHOG0485, cHOG0512, cHOG0543, cHOG0560, cHOG0572, cHOG0579, cHOG0690, cHOG0700, cHOG0703, cHOG0737, cHOG0743, cHOG0745, cHOG0752, cHOG0753, cHOG0757, and cHOG0772), 21 30S ribosomal subunit clusters (cHOG0154, cHOG0271, cHOG0274, cHOG0379, cHOG0396, cHOG0539, cHOG0564, cHOG0655, cHOG0660, cHOG0675, cHOG0680, cHOG0692, cHOG0709, cHOG0726, cHOG0740, cHOG0750, cHOG0758, cHOG0760, cHOG0770, cHOG0771, and cHOG0774), and 13 amino-acyl tRNA synthetase clusters (cHOG0160, cHOG0184, cHOG0199, cHOG0250, cHOG0289, cHOG0306, cHOG0435, cHOG0468, cHOG0484, cHOG0487, cHOG0514, cHOG0536, and cHOG0672). In addition, we identified 11 cHOGs as containing RNA polymerase II-like enzymes (cHOG0165, cHOG0338, cHOG0407, cHOG0412, cHOG0492, cHOG0507, cHOG0679, cHOG0722, cHOG0741, cHOG0773, and cHOG0779), two containing origin recognition complex homologs (cHOG0234 and cHOG0244), three containing histone acetyltransferases (cHOG0049, cHOG0352, and cHOG0398), two containing transcription initiation factor IIB homologs (cHOG0004 and cHOG0018), and one containing transcription initiation factor IID homologs (cHOG0044).

(ii) An additional 10% of cHOG protein clusters was classified as being involved in cellular processing and signaling. Half of the proteins in these cHOGs were involved in posttranslational modification, protein turnover, or assembly, including two proteasome subunit clusters (cHOG0058 and cHOG0127), four heat shock protein clusters (cHOG0150, cHOG0156, cHOG0458, and cHOG0678), and two thermosome subunit clusters (cHOG0320 and cHOG0344). Three categories of COGs, nuclear structure, cytoskeleton, and extracellular structure, were not represented in any of the HOGs.

(iii) The largest number of cHOGs (33%) was classified as being involved in metabolism. Unlike cHOGs involved in information transfer and storage and cellular processes and signaling, there was no single category of metabolism that was overwhelmingly abundant. Four categories, energy production and conversion, amino acid transport and metabolism, nucleotide transport and metabolism, and coenzyme transport and metabolism, each contained over 40 cHOGs and accounted for 5% or more of the core clusters. Included in these cHOGs were nine ATP synthase subunit clusters (cHOG0124, cHOG0195, cHOG0233, cHOG0293, cHOG0302, cHOG0527, cHOG0600, cHOG0616, and cHOG0909), and ten NADH:ubiquinone oxidoreductase subunit clusters (cHOG0036, cHOG0126, cHOG0132, cHOG0187, cHOG0381, cHOG0453, cHOG0496, cHOG0599, cHOG0775, and cHOG0777).

The number of cHOGs associated with a cellular process did not necessarily correlate with the degree of conservation of that process. In particular, while there was a smaller number of cHOGs associated with information transfer and storage than metabolism, the proteins involved in information transfer and storage were more conserved in haloarchaea than those of metabolism or cellular processing and signaling. A large majority (65%) of HOGs associated with information transfer and storage was conserved in all nine genomes, whereas only 46% and 38% of the metabolism and cellular processing and signaling HOGs, respectively, were conserved in all of the genomes.

### Newly sequenced haloarchaeal genomes

We also used BLAST analysis to determine if the cHOG proteins were conserved in four recently completed genomes (Table 4; see also Additional file 1). Homologs of the overwhelming majority of the cHOGs (784 out of 799) were identified in the recently completed genomes of *Haloterrigena turkmenica*, *Halopiger xanaduensis*, *Natrialba magadii*, and *Halalkalicoccus jeotgali*, with only six, two, five, and six clusters absent in these species, respectively (Table 4). Among the unique genes, five out of 60 tucHOGs and one of the 29 ucHOGs in the nine original genomes analyzed were absent in one or more of the four newer haloarchaeal genomes (Table 4).

**Table 4:** Haloarchaeal protein clusters (HOGs) identified with nine and 13 genome data sets.

	No. of clusters with original 9 genome data set	No. of clusters removed from each category	No. of clusters added to each category	No. of clusters with 13 genome data set
cHOGs	799	15		784
cHOGs associated with COGs	288	6^a^		282
cHOGs associated with COGs & KOGs	422	3^b^		419
ucHOGs	89	6		83
nucHOGs	29	1^c^		28
tucHOGs	60	5^d^		55
aHOGs	3656		15	3671
aHOGs associated with COGs	409		6	415
aHOGs associated with COGs & KOGs	259		3	262
aHOGs with no associated COGs or KOGs	2988		6	2994

^a^- Homolog for HOG0026 not identified in *H. jeotgali*. Homologs for HOG0069, HOG0288, and HOG1012 not identified in *N. magadii*. Homologs for HOG0231 and HOG0408 not identified in *H. turkmenica*.

^b^- Homologs for HOG0166 and HOG0221 not identified in *H. turkmenica*. Homolog for HOG0518 not identified in *H. jeotgali*.

^c^- Homolog for HOG0305 not identified in *H. jeotgali*.

^d^- Homologs for HOG0048, HOG0714, and HOG0735 not identified in *H. jeotgali*. Homologs for HOG0120 and HOG0905 not identified in *H. xanaduensis*, *N. magadii*, or *H. turkmenica*.

## Discussion

Our current study has established core and unique haloarchaeal proteins and assigned likely functions to these conserved haloarchaeal proteins among sequenced haloarchaea. The core haloarchaeal orthologous groups (cHOGs) contained nearly 800 protein clusters that accounted for 21 - 33% of each predicted haloarchaeal proteome. The majority (89%) of the core proteins could be assigned specific or general functions based on association with NCBI KOGs and/or COGs, while the remainder (11%) were novel and could not be correlated to any previously known protein clusters. Based on further analysis of four recently sequenced haloarchaeal genomes and statistical analysis of alignments with non-haloarchaeal homologs, 55 protein clusters (named tucHOGs) were identified as haloarchaeal signature proteins.

The precise functions of the signature proteins are not clear because of their unique nature and the dearth of experimental studies focused on these genes. Only a single example among the truly unique haloarchaeal orthologous groups, Ral (tucHOG0456), was examined in any previous experimental work and was suggested to function in double-stranded DNA break repair and desiccation/radiation tolerance in the model haloarchaeon, *Halobacterium *sp. NRC-1 [27]. Transcriptome analysis of both UV irradiated *Halobacterium *sp. NRC-1 and its highly ionizing radiation resistant mutants showed an up-regulation of the *rfa3*-*rfa8*-*ral *operon, consistent with their involvement in DNA repair and protection [27]. Due to the transcriptional linkage of the three genes, and the presence of oligonucleotide binding (OB) folds in *rfa3 *and *rfa8*, the *ral *gene was also hypothesized to function as part of the eukaryotic-type single-stranded DNA binding RPA complex. However, analysis of the amino acid sequence of Ral did not reveal an OB fold domain, and it is not clear whether it serves as the third subunit of the RPA complex, replacing the RPA14 subunit found in eukaryotic organisms. While additional experimental studies are still required to determine the precise function of Ral, the possibility that it, as well as those of the other uniquely conserved haloarchaeal proteins, functions in adaptation of these organisms to their naturally extreme environments is an attractive hypothesis.

A somewhat larger (83) group of protein clusters, unique core haloarchaeal proteins (ucHOGs), includes 28 members which are nearly unique to haloarchaea (nucHOGs) and 55 which are truly unique to haloarchaea (tucHOGs). Our bioinformatic analysis of the ucHOGs suggested that they are quite typical of haloarchaeal proteins in pI, molecular weight, and GC-composition of their genes. The average pI of the ucHOGs is 4.7, similar to other haloarchaeal proteins (see Additional file 2). Similarly, the average G + C content of the ucHOGs are typical for each haloarchaeal chromosome (ranging from 68.5% for *Halobacterium *sp NRC-1 to 48.0% for *H. walsbyi*) (see Additional file 3). Their average molecular weight, 19.8 kDa, is somewhat smaller than predicted haloarchaeal proteins in general, 31 kDa (see Additional file 4). Their smaller size is consistent with their role as accessories to protein complexes, as suggested for the Ral protein in single-stranded DNA binding and DNA repair and protection. For example, as a group, ucHOGs may improve the activity or function of complexes in the cytoplasm with essentially saturating concentrations of KCl [10]. The great majority of ucHOGs appear to be soluble proteins (unpublished data).

The genomic distribution of ucHOG protein genes was examined and they were found to map overwhelmingly on the chromosomes in all of the haloarchaeal microorganisms (see Additional file 1). In the case of *Halobacterium *sp. NRC-1, all of the tucHOGs and all but one of the nucHOGs were located on the chromosome (Figure 4). The haloarchaea do not contain more than one or at most two of these proteins on megaplasmids. These findings suggest that the ucHOG proteins serve integral functions in these microorganisms and are likely important and possibly critical for survival. In addition to their likely important function, the ucHOGs, and especially the signature proteins (tucHOGs) and their genes, will also be useful as markers for the presence of members of the Haloarchaeaceae family in the environment.

Of the 83 ucHOGs, 28 were not completely unique to haloarchaea, with one or a few homologs present in non-haloarchaea (see Additional file 1). A large fraction (46%) of the hits were to methanogenic Archaea belonging to the Methanosarcinaceae, Methanosaetaceae, and Methanocellaceae families, which are relatively close to haloarchaea based on phylogenetic analysis of 16S sequences and include some moderate halophiles [1]. There were also a number of hits to halophilic bacteria, e.g., *Salinibacter ruber*, which may be the result of lateral gene transfer between species in a common environment [10]. Of the clusters determined to not be uniquely haloarchaeal, 14 were associated with archaeal COGs (arCOGs) containing non-haloarchaeal homologs, consistent with their presence in more than a single family of Archaea [28] (see Additional file 1). This may reflect the distinct and common ancestry of the Archaea.

Prior to our study, an analysis of conserved proteins in the Archaea was first completed on eight archaeal genomes which did not include any haloarchaeal genomes [29]. In this early study, 351 signature proteins present in at least two of the archaeal genomes were identified. In a subsequent study, 11 archaeal genomes were compared, including two haloarchaeal genomes [30]. The number of signature proteins shared by all 11 genomes decreased to only six and an additional 30 were identified in the majority of archaeal genomes. In an analysis of four haloarchaeal genomes, 127 haloarchaeal-specific proteins were reported [30]. Of these, we classified 51 as signature proteins or tucHOGs, 13 as nucHOGs, while the remaining 63 were either missing in one or more of the 13 haloarchaeal genomes or were associated with a COG (see Additional file 5). In another report, ten haloarchaeal genomes were recently compared and 112 'signature' clusters were reported [19], of which we found that 50 were similar to tucHOGs and 11 are like nucHOGs (see Additional file 6).

Several studies aimed at identifying signature proteins in other taxonomic groups have been conducted for organisms from other domains of life. Among bacteria, an analysis of actinobacterial genomes found 29 signature proteins present in the majority of genomes and an additional 204 that are found in some, but not all of the genomes [31]. In another study [32], five Chlamydial genomes and one Parachlamydial genome were compared, and 59 proteins were conserved in all six genomes, coded by hypothetical genes with no known functions. Two subsequent studies of α-proteobacterial genomes reported signature proteins [33,34]. Initially three genomes were compared and six signature proteins were identified in the majority of α-proteobacterial genomes and an additional 47 proteins were identified in some but not all subgroups [33]. With the increase to 12 α-proteobacterial genomes, further work showed that only four of the original six signature proteins were present in all of the genomes [34]. Among eukaryotes, 300 conserved signature proteins were identified in sequenced genomes, including the deeply branching *Giardia lamblia *species [35-37].

The entire set of genes within a given species or group of organisms, in essence, the combination of the core and all dispensable genes, is sometimes referred to as the "pan-genome" [38]. With this approach, as more whole genomes become available, the size of the pan-genome increases due to an increase in the number of accessory genes, while the size of the core-genome asymptotically reaches a minimum. While there are numerous studies of species level pan-genomes, there are only a few published studies at the genus or family level. A study of 26 genomes from the *Streptococcus *genus found that the core-genome contains 611 orthologous groups, which constituted 26 - 30% of any one genome [39]. Analysis of 11 genomes from the Vibrionaceae family found the core-genome of 1,882 orthologous groups constituted 32 - 50% of these genomes [40]. Analysis of six genomes from the Enterobacteriaceae family identified 2,125 core orthologous groups that accounted for 43 - 88% of these genomes [41].

Our result from this study of the Haloarchaeaceae family showed that 21 - 33% of each genome constituted the core-genome and was similar to the results reported in earlier studies on other groups. Moreover, the great majority of core orthologous groups identified in the first nine haloarchaea were conserved in the subsequent four sequenced species. Our preliminary results with analysis of the pan-genome of haloarchaea show an expanding number of dispensable genes among members of this group (data not shown). The sequencing of additional haloarchaeal genomes and metagenomes and further bioinformatic analysis are likely to yield additional insights into the genetic composition of this interesting group of extremophilic microorganisms [42].

## Conclusion

The signature and core genes and proteins are valuable concepts for understanding phylogenetic and phenotypic characteristics of coherent groups of organisms. Our analysis of 13 haloarchaea from different genera has established that the haloarchaeal proteome consists of 4,455 orthologous groups (HOGs), 784 of which form the core proteome (cHOGs), and 55 of which constitute haloarchaeal signature proteins (tucHOGs). The conservation of the cHOG and tucHOG clusters suggests that they may be essential or vital for survival. An attractive hypothesis, similar to what has been suggested for Ral, the only tucHOG with a predicted function, is that these small, chromosomally encoded proteins may act as accessory proteins enhancing macromolecular function in extreme conditions.

## Methods

### Sources of nucleotide and protein sequences

Nucleotide and protein sequences were obtained for completed haloarchaeal genomes from NCBI: *Halobacterium *sp. NRC-1 ATCC 700922 (NRC-1) [8], *Haloarcula marismortui *ATCC 43049 (Hma) [43], *Natronomonas pharaonis *DSM 2160 (Nph) [44], *Haloquadratum walsbyi *DSM 16790 (Hwa) [45], *Halorubrum lacusprofundi *ATCC 49239 (Hla) [46], *Halogeometricum borinquense *DSM 11551 (Hbo) [47], *Halomicrobium mukohataei *DSM 12286 (Hmu) [48], *Halorhabdus utahensis *DSM 12940 (Hut) [49], *Haloferax volcanii *DS2 (Hvo) [50], *Haloterrigena turkmenica *DSM 5511 (Htu) [51], *Natrialba magadii *ATCC 43099 (Nma) [52], *Halalkalicoccus jeotgali *B3 (Hje) [53], and *Halopiger xanaduensis *SH-6 (Hxa) [54].

### Construction of protein clusters

For the initial nine genomes, we used the method of Tatusov [23,24] to determine best reciprocal hits and the program MUSCLE for multiple sequence alignments [55]. Conserved protein clusters were used to construct orthologous groups using in-house Perl scripts and manual navigation of data stored in a MySQL database and served on our Linux-Apache servers (HaloWeb - http://halo4.umbi.umd.edu) [56]. Subsequently, we analyzed four additional sequences using our HOGnitor, via BLAST analysis. Similar non-haloarchaeal proteins were identified with BLAST analysis using HOG proteins as query sequences against the NCBI non-redundant database (June 5, 2011 version).

### Statistical analysis of proteins clusters

Significance of protein assignment to clusters was established by base composition-preserved randomized pairwise global alignments using the method of Needleman and Wunsch [26,57]. Scores of paired alignments were compared to scores and standard deviation for 50 randomized sequences with base composition-preserved. Protein families displaying greater than 99.9999% confidence were grouped into haloarchaeal orthologous groups (HOGs), and families with similar non-haloarchaeal proteins displaying greater than 99.0% confidence were grouped into nearly unique haloarchaeal orthologous groups (nucHOGs) [25,58].

### Correlation with COGs, KOGs, and arCOGs of haloarchaeal orthologous groups and functional classification

Haloarchaeal orthologous groups or HOGs were correlated with prokaryotic (COGs) and eukaryotic (KOGs) orthologous groups at NCBI using one of three methods: (1) HOGs were correlated to COGs using the *Halobacterium *sp. NRC-1 COGs as reference [23,24]. (2) COGs and KOGs were correlated based on the *Saccharomyces cerevisiae *predicted proteins. (3) HOGs associated KOGs were also identified using the KOGnitor tool [24]. HOGs were correlated with the clusters of archaeal orthologous groups (arCOGs) based on *Halobacterium *sp. NRC-1 proteins [28].

### Genomic and protein analysis

Genomic analysis was conducted using tools available on our HaloWeb servers [56]. Protein analysis was carried out using either stand-alone Perl scripts or Perl scripts running the Wisconsin Package protein analysis programs [59]. Chromosome maps were generated using either our HaloWeb servers or GenomeVx software [56,60].

## Competing interests

The authors declare that they have no competing interests.

## Authors' contributions

MDC, PD, and SD contributed to the bioinformatic analysis and writing the manuscript. All authors read and approved the final manuscript.

## Supplementary Material

Additional file 1 **Core haloarchaeal orthologous groups (cHOGs) proteins, associated COGs, KOGs, and arCOGs, genomic location, and confidence levels**. Click here for file

Additional file 2 **Statistical values for pI of haloarchaeal proteomes and ucHOGs**. Click here for file

Additional file 3 **Statistical values for G + C composition of haloarchaeal chromosomes and ucHOGs**. Click here for file

Additional file 4 **Statistical values for molecular weight of haloarchaeal proteomes and ucHOGs**. Click here for file

Additional file 5 **HOG association with 127 haloarchaeal specific proteins **[30]. Click here for file

Additional file 6 **HOG association with 112 haloarchaeal signature clusters **[19]. Click here for file
